# Care Compromised: Cases from a Jail Medical Ward

**DOI:** 10.1007/s11013-025-09902-x

**Published:** 2025-02-22

**Authors:** Liza Buchbinder

**Affiliations:** UCLA Center for Social Medicine and Humanities, B7-435, UCLA Semel Institute, Los Angeles, CA 90095-1759 USA

## Introduction

The COVID19 pandemic was an unprecedented period of touchlessness. Social distancing was a public health mantra, and doctors and nurses were precluded from physically interacting with patients. This was a temporary suspension of usual practice. But deviations from usual practice are the status quo in American jails. County and city governments across the country continue to build jails with medical wards to fulfill the ideal of carceral humanism by establishing a safety net within corrections. (Kilgore, 2014; Norton et al., 2024).

Despite a trend toward more humane approaches to incarceration with resources funneled toward greater space, increased social services, and expanded staffing, challenges persist. Local governments need to be cognizant of the inherent contradictions and tragic limitations of jail-based medical treatment. As a provider for a large county jail in southern California, I have witnessed firsthand how constricted resources, mutual distrust, security protocols, and the stigma of criminality permeate everyday interactions. In the medical ward where I worked, clinical norms were superseded by jailhouse strictures. The ward was designed for complex but medically stable patients and the ward’s providers come from different enterprises, including private contractors and salaried government workers whose roles crisscrossed through the layers of medical services, from urgent care to specialty clinics.

Within the carceral setting, legal scholars have historically problematized touchlessness as it relates to the “cruel and unusual punishment” of solitary confinement, where long-term isolation can lead to “social death” (Lisa Guenther, 2013, p. 3), an unraveling of personhood (Ibid., Myers, 2012; Rhodes, 2004), and the incapacity to function socially upon release (Reinert, 2017). In the jail medical ward, inmates lived in *de facto* solitary confinement because they were administratively separated from the rest of the population based on their pathologies. In addition to the psychic harms of isolation, there were patients who received compromised care in a jail instead of a clinic or hospital. As counties increasingly build jail medical wards across California (Lamb & Weinberger, 2005; Norton et al., 2024), administrators should be realistic about the quality of care they can provide behind bars.

While some may argue that jails and prisons provide inmates with healthcare they would not receive out on the streets (Marks & Turner, 2014; McDonnell et al., 2014), my experience seeing patients through heavy metal doors and onerous security protocols suggests that barriers were so great that often the only way to treat a patient was to transfer them out. This led to a ‘hot potato’ dynamic as providers in the jail and hospital transferred high-risk patients back and forth out of fear that a bad outcome would occur while under their watch.

This was the case for Lydia, a young woman who had an aortic aneurysm threatening to rupture. She refused surgery because she wanted a second opinion. As jail providers, we felt obligated to transfer her to the hospital every time she described chest pain, regardless of the intensity. The emergency physicians at the hospital would then order a CT scan that was inevitably negative. Then, within hours, Lydia was back to her cell until her next bout of angina—waiting, ostensibly, for the worst to happen before she could be monitored in a safe environment.

Other cases also illustrated how the constraints on therapeutics of working in a penal institution created a near-impossible environment to properly care for patients. For Mark, the problem was touchlessness. He was afflicted with lower leg cellulitis (soft tissue infection) and was taking oral antibiotics to treat it. Per written and verbal reports, he was suicidal, belligerent, and had shattered his glass cell door during a heated outburst. As I approached Mark’s cell during rounds, I felt threatened for my personal safety despite the accompaniment of an officer who had the authority to use “objectively reasonable” force [while firearms are prohibited, jail officers could use handcuffing, hobbling, leg restraints and capsicum spray to subdue inmates (Lamb & Weinberger, 2005; Norton et al., 2024; Sussman, 2011, p. 1376)]

Mark’s mental and physical illness put him at high risk of falling victim to jail-based violence. Despite knowing this, I was still afraid. When I approached Mark’s door during my rounds, he did not threaten me; instead, he predictably raised the cuff of his pants to reveal his ankle. I stooped down to the bottom of the door and pressed close to the cloudy glass for a better look. From what I could see, his leg was weeping, red, and swollen. A close physical examination would have allowed me to assess the character and extent of his infection, but from what I could appreciate across the glass, the pills were not working. Given the risks of undertreatment, I ordered an intravenous antibiotic. The bedside nurse said that she could not give the medication because plastic tubing was a suicide risk, and chemical or physical restraints were prohibited. While restraints to support security and incarceration—such as handcuffs—were commonplace in the jail, they were not allowed for medical purposes. The only solution for Mark was painful intramuscular injections or transfer from the jail to the hospital, where more equipment, personnel—and the use of restraints—were available. Uncomplicated cellulitis is a ‘bread and butter’ diagnosis with a straightforward treatment algorithm under typical conditions (Sullivan & de Barra, 2018). Yet, the jail was not a typical setting in which to deliver medical care. Due to my personal fear and the jailers’ concerns, I opted not to palpate the wound in order to determine the appropriate antibiotics. Since Mark was also on suicide watch, we could not provide him standard of care on the inside (Chui, 2018) and had to transfer him out for intravenous antibiotics. While Mark’s situation is particular to him, each patient/inmate had his/her own combination of health needs and institutional limitations that perpetuated a system of routinely compromised care.

## Lessons from Phenomenology: How Touchlessness Compromises Care

Beyond a better diagnosis, the barriers to touch in Mark’s case denied us the possibility of a more meaningful therapeutic alliance. Touch in medicine has historically been valued as a data gathering tool via the physical examination (Hyman, 2020), or a professionalized form of communication that reassures patients and engages doctors in a therapeutic alliance (Hyman, 2020; Verghese, 2016). Drawing on the phenomenological concepts of embodiment, intersubjectivity, and empathy, I argue instead that the impact of touch is more extensive than these conventional explanations, and the endemic scarcity of touch in the jails compromises care. Rejecting an atomized dualism that separates the mind and body or subject and object, phenomenologists conceptualize human experiences as “more dynamic, intersubjective, and pleural… [than can] be referenced by the singular term ‘the body’” (Lock & Farquhar, 2007, p. 2). In short, there is a holism to the social body that is incompletely understood through medical science.

Embodied engagement is a related term and speaks to the way in which the body is a medium of consciousness for perceiving the world. French phenomenologist Maurice Merleau-Ponty wrote about embodied actions and the way in which one’s body is intimately intertwined with one’s perception of other people and the environment (Merleau-Ponty, 2013). Akin to an athlete in flow where the boundaries between the subjective experience and objective reality blur (Dreyfus, 2007), the seasoned doctor with her cultivated exam skills over years of training and thousands of patient visits, comes to the interaction with a holistic perception that is integrally tied to her body and allows for an openness to uncertainty and mutual vulnerability (Lock & Farquhar, 2007). Unfortunately, in the jail, mutual vulnerability leads to mutual threat. Steel doors, fear, and security mandates preclude an openness to uncertainty, or a “curiosity” about “patients’ distinct worlds” (Halpern, 2001, p. 133). With this closure comes a loss of empathic opportunity. Yet, empathy is a core component of good doctoring and greater physical barriers strip the encounter of the necessary conditions for embodied engagement and openness. While aspirational, it is not easy to eschew security measures. An encounter with patient Jones is a case in point.

## Jones’ Gesture

Confident that I could be effective in connecting with patients in the jail, I was adamant about speaking to Jones face-to-face about his Hepatitis C. Despite a known diagnosis, he was refusing curative medications. I assumed the root of his reluctance was due to busy and inured staff neglecting to explain how deadly Hepatitis C can be and how simple it is to treat.

In response to my request for a security chaperone during rounds, a much larger group of officers accompanied me than usual. They gathered outside his cell. Before unlocking the door, the leader of the unit asked for my confirmation that entering was medically necessary. I reiterated the importance, and he opened the door. The lead officer followed standard protocol by telling Jones to stay seated on the bed. When I entered, I took Jones to be in his early forties, well-built, and healthy despite his years of incarceration. He avoided eye contact as I talked and begrudgingly responded to my efforts at conversation. It was clear that he was not interested in discussing liver failure. At a certain point, he switched his weight onto his left side and made a motion to rise. Suddenly, the lead officer stepped in and said, “Jones, sit down…. Dr Buchbinder, I need to speak to you.” I stepped back into the hallway and the door quickly closed behind me. “I thought you said that you needed to physically examine the inmate. You were just talking,” he said. “Yes, I needed to talk to him face to face about his medical condition,” I said. The officer went on to explain that this patient was classified as K10 (high security risk) and had a history of assaulting staff. If anything happened to me while I was in the cell, he would be liable for allowing my entry without an appropriate reason. If I was harmed, he would have to answer to his superior and his team would be cited. Confused at first and then grateful for the protection, I took some time to process the situation. I was not sure if Jones really meant to stand up or if he just made a misplaced gesture, but there was little room for error. In the end, I walked away from the encounter feeling ashamed by my arrogance and more appreciative of those whose job it is to protect me.

In addition to the physical separation from staff, the inmates on the medical ward were largely sequestered from each other. During my shifts, I rarely saw them physically interacting unless housed in a rare, shared cell. The only connection with the outside came from a transfer out to the hospital, a bus ride to court, or a phone call via the shared telephone that was available for a limited time each day. Patients also left the medical ward if they were downgraded to another form of housing, like ‘diabetic housing’ or ‘general population.’ As the vignette about Jones demonstrates, clinical exams were limited to low-risk patients, while others are seen across the glass. A doctor, nurse practitioner, or physician assistant would round on each ward patient every other day, and the nurses would come twice daily to distribute medications. Custody officers saw the inmates most frequently to scan the room doors during their regular bed checks. On these occasions, an inmate may have scored a snippet of conversation if they happened to capture the passing officers’ attention. Alternatively, they were allowed to yell across the hallway in the hopes of conversing with a neighboring inmate.

Phenomenology’s emphasis on the primacy of perception and the body-as-lived compared the scientific understandings of the biological body also attends to the contestations over the meaning of the clinical encounter. As Halpern writes, power dynamics, social structures, and cultural divisions mediate the doctor-patient dyad—and there are others who are also embroiled in the encounter. In the case of the jail medical ward, those other people include correctional officers and nurses (Halpern, 2001).

## The Multiplicity of Touch Resonances

Jail medicine can also be challenging because patient adherence is inconsistent, and this inconsistency is shaped by contestations over the meaning of the clinical encounter. The meaning of touch and its appearance to the inmate may be different from the way the provider perceives it. To the inmate, most touch is punitive, involving handcuffs, restraints, and the possibility of further punishment if a jailer misconstrues a certain gesture as threatening. Moreover, handcuffs reinforce inmate subordination. As physician-anthropologist Carolyn Sufrin writes, “Correctional facilities are not designed to treat illness, but to punish and confine” (Sufrin, 2017, p. 59). Tasked with helping patients get better, my role was at odds with the mission of county corrections. And the barriers mediating my capacity for embodied engagement with inmates—including my own fears for personal safety—served as a reminder of the contradictions of jailcare.

Just as security takes precedence over clinical care, so too do the inmate’s legal priorities. For each inmate, the set of circumstances is different. While unknown to the provider, these circumstances may dictate strategic jockeying for or against treatment. Some inmates wanted to stay in the ward, while others wanted out. Some benefited from their diagnosis, while others were harmed, and this often was related to whether a particular court date or transfer schedule was advantageous or disadvantageous. As a result, inmates frequently refused treatment as a means of asserting control, or because physical well-being was often a lower priority to their legal woes. During the pandemic, inmates would also withhold symptom reports given concerns about lockdowns, missed court dates, and increased jail time.

Trust that correctional staff wanted to help rather than harm was a leap of faith given the overarching mission of the jail to contain and punish. The meanings attributed to touch also differed depending on who was involved. For an inmate, a provider’s palpation of a lymph node in the neck may trigger associations with the everyday forms of touch in the jail, including handcuffing and ushering from one location to another. An inmate’s gesture in touching back may also be misconstrued as threatening, heightening the dangers of touch encounters. As such, touch was often a means of reinforcing inmate subordination rather than a medium for healing and empathy.

## Shared Humanity Under the Glare

Despite these challenges, moments of connection did occur. I witnessed this during a late-night transfer of a patient to the county hospital when an experienced jail provider showed compassion that verged on the intimate. An elderly inmate named Frank had symptomatic anemia and was upset that he needed to be handcuffed to a portable gurney for transport to the hospital for a transfusion. Frank was crying out, “I’m not an animal. You’re treating me like I’m an animal.” The young correctional officers were unsure how much force to use vis-à-vis a defiant, elderly man. As two paramedics watched from outside of the cell, they were having trouble getting the man to comply.

Eventually, a seasoned nurse arrived. “Don’t worry, I’ll talk to him,” he said. Originally from southeast Asia, the nurse had worked the night shift for fifteen years. He preferred the nights to days because the lighter traffic cut down on his two-hour commute. Frank, the man he was tasked with convincing, was white, disheveled, and in his late 60s. He was frail with shoulder length hair matted to his scalp. He wore the jail uniform of a white shirt, brown scrubs, and white socks. No shoes were allowed. The nurse entered Frank’s cell and crouched down next to him. As a pleading gesture, he touched Frank’s arm and asked him to be “reasonable” and take the cuffs. Like Frank, the nurse was also logging in long hours under the incessant glare of the ward’s florescent lights. While he could leave at any time, in that moment, they were in it together. The nurse tried to convince him that it was just a formality, not an insult. He promised that the officers would not even be cuffing both his wrists. They would just cuff one wrist to the siderail. His tone conveyed a shared understanding that the restraint protocol was unfortunate, but necessary. Finally, Frank acquiesced. He went onto the gurney and the custody agent quickly cliqued the handcuff. As they walked away, the officer turned back to the nurse to thank him.

Touch was not a diagnostic tool in this situation. But the corollary to touch—embodiment and its recognition—was certainly at play. With respect to the nurse, Frank, the handcuffs, and the gurney, this instance of compassion was initiated with the nurse’s attentive gestures of recognition that temporarily violated the anesthetic distance integral to this inmate’s imprisonment. The nurse’s assertion of embodied presence via touch was transgressive while also essential to Frank’s compliance.

## Conclusion

In all these cases, the limits of jailcare played out in different ways. For Frank who did not want to be handcuffed, the nurse’s physical presence—his haptic signaling, by adapting himself to Frank’s position—encouraged him to comply and accept humiliating treatment for an escalation of care. For Mark, the issue of touching or not touching was about diagnosis but was also an indicator of how difficult it is to provide treatment within the convoluted morass of multiple bureaucratic protocols. The lack of touch in the encounter also precluded the possibility for empathic vulnerability, hampering the possibility for a deeper therapeutic alliance. For Jones, my inability to counsel because of the heightened scrutiny of his movements in light of his security level and the officers’ liability was a stark reminder that this is not just about the doctor and patient engaging in a decontextualized physical encounter. Rather, these encounters were imbued with meanings that resonated far beyond cell doors where glaring white lights, surveillance cameras, and security protocols beset intersubjective carceral engagements. To be a healer and simultaneously one of the many manifestations of violent control are the paradoxes of practicing jail-based care. The providers’ necessary reliance on the glass comes with an understanding that this care is compromised.

Compromised care at a massive scale is increasingly becoming a reality as carceral populations expand. On November 1, 2024, California voters approved Proposition 36, which is an initiative to effectively bring back the 1994 Three Strikes Law that led to unconstitutional levels of overcrowding and thousands of mandatory sentences of 25 years to life for petty crimes. Now that Proposition 36 passed, jail and prison populations will balloon in size (Vera Institute of Justice, 2024), and the majority of felony convictions under the new law will be for non-violent drug offenses or retail crimes. Jails and prisons cannot deliver comparable medical care to community-based clinics and hospitals, but they have become the de facto safety net for hundreds of thousand Californians. Rather than siphon money from community-based and county treatment programs as the costs of expanded incarceration rise, state and county governments should recognize—as the patients in this article demonstrate—that there are real structural challenges to providing adequate care behind bars. Instead, policymakers should focus resources on keeping people out of the correctional system, and there is precedent for doing so. LA County’s Whole Person Care program, which ran from 2016 to 2021, was an exemplary initiative dedicated to caring for the county’s sickest and most vulnerable residents by diverting low-level offenders from jails to primary care clinics and long-term housing. The political establishment needs to tout these gains rather than look for tough-on-crime solutions that will result in more sickness for justice-involved individuals and higher costs to the public.
